# Risk Factors for hypospadias in Northwest Russia: A Murmansk County Birth Registry Study

**DOI:** 10.1371/journal.pone.0214213

**Published:** 2019-04-04

**Authors:** Anton A. Kovalenko, Tormod Brenn, Jon Øyvind Odland, Evert Nieboer, Alexandra Krettek, Erik Eik Anda

**Affiliations:** 1 Department of Community Medicine, UiT -The Arctic University of Norway, Tromsø, Norway; 2 International School of Public Health, Northern State Medical University, Arkhangelsk, Russia; 3 Department of Biochemistry and Biomedical Sciences, McMaster University, Hamilton, ON, Canada; 4 Department of Biomedicine and Public Health, School of Health and Education, University of Skövde, Skövde, Sweden; 5 Department of Internal Medicine and Clinical Nutrition, Institute of Medicine, Sahlgrenska Academy at University of Gothenburg, Gothenburg, Sweden; University of Missouri Columbia, UNITED STATES

## Abstract

**Background:**

Hypospadias is the most common congenital anomaly of the penis, but its causes are mainly unknown. Of the risk factors identified, the most plausible are hormonal and genetic. The aim of this study was to identify risk factors for hypospadias in Northwest Russia based on registry data.

**Methods:**

The study population included male infants registered in the Murmansk County Birth Registry between 1 January 2006 and 31 December 2011 (n = 25 475). These infants were followed-up for 2 years using the Murmansk Regional Congenital Defects Registry to identify cases of hypospadias not diagnosed at birth. We used logistic regression analysis to examine the contributions of hypospadias risk factors.

**Results:**

Out of 25 475 male infants born during the study period, 148 had isolated hypospadias. The overall prevalence rate was 54.2 (95% CI 53.6–54.8) per 10 000 male infants. Those born to mothers with preeclampsia (OR = 1.65; 95% CI 1.03–2.66) or infant birthweight < 2500 g (OR = 2.06; 95% CI 1.18–3.60) exhibited increased risk for hypospadias. Maternal age, smoking during pregnancy, folic acid intake during pregnancy or hepatitis B surface antigen positivity did not associate with increased risk of hypospadias.

**Conclusions:**

Combining data from a birth registry with those from a congenital defects registry provided optimal information about the prevalence of hypospadias and its association with low infant birthweight and preeclampsia. These factors have in common changes in hormone levels during pregnancy, which in turn may have contributed to hypospadias development.

## Introduction

Hypospadias is a male-specific congenital birth defect that leads to displacement of the external urethral orifice and often associates with an incomplete development of the foreskin [1]. It usually develops 8–14 weeks after conception and is one of the most common structural malformations in humans. It occurs in 18.6 per 10,000 newborn[2]. A single cause of hypospadias is still not identified, although it appears to be a combination of genetic factors and mother’s exposure to endocrine disruptors [3]. Cases of this defect are usually relatively mild, but when severe may constitute a symptom of a disorder of sexual differentiation [4]. The prevalence of hypospadias increased in many countries during the 1960s to early 1990s [5]. This trend could be due to an actual increase of hypospadias’ events or improved diagnostic practices [5]. Recent reports show that the prevalence in most countries has not increased much since the mid-1980s [6] and has been stable during the 2001 to 2010 period in 23 EUROCAT (European network of population-based registries for epidemiologic surveillance of congenital anomalies) registries [2].

The standard classification of hypospadias is based on the location of the urethral meatus; namely distal, midshaft, or proximal [7]. Most infants with hypospadias are diagnosed soon after birth while still in the hospital. However, slight displacement of the urethral opening may be subtle and more difficult to identify.

Over the past 30 years male reproductive health has changed; specifically, sperm counts have decreased and the number of cases of undescended testes and testicular cancer have increased [8]. This has prompted scientists to investigate the possible role of environmental contaminants, especially those with endocrine-disruption capabilities [9]. The cause of most hypospadias cases remains unknown, including the potential impact of genetic and environmental factors. Nevertheless, several plausible associations have been suggested [10]. Among these are advanced maternal age, increased body mass index (BMI) of the mother, preexisting diabetes, preeclampsia during pregnancy, smoking, phytoestrogens intake during pregnancy, exposure to different chemicals, and some infectious diseases such as hepatitis [11–16].

A retrospective study published in 2006 was the first to investigate possible risk factors for hypospadias in the Murmansk Region. It reported no negative effect of maternal exposure to water-soluble nickel compounds on the risk of genital malformations in the offspring of pregnant women in the town of Monchegorsk [17]. This cohort included women who worked in the local nickel refinery complex. The prevalence rate of hypospadias in Murmansk County has remained unusually high at 25.7 per 10,000 newborns during 2006–2011, compared with those for Arkhangelsk County (4.1 per 10,000 newborns) and Norway (13.0 per 10,000 newborns) during the same period [18]. In the current article, we combine pertinent information available from the Murmansk County Birth Registry (MCBR) and the Murmansk Regional Congenital Defects Registry (MRCDR) in order to explore potential risk factors that may help explain the high occurrence of hypospadias in Northwest Russia.

## Materials and methods

### Study population

**Fig 1:**
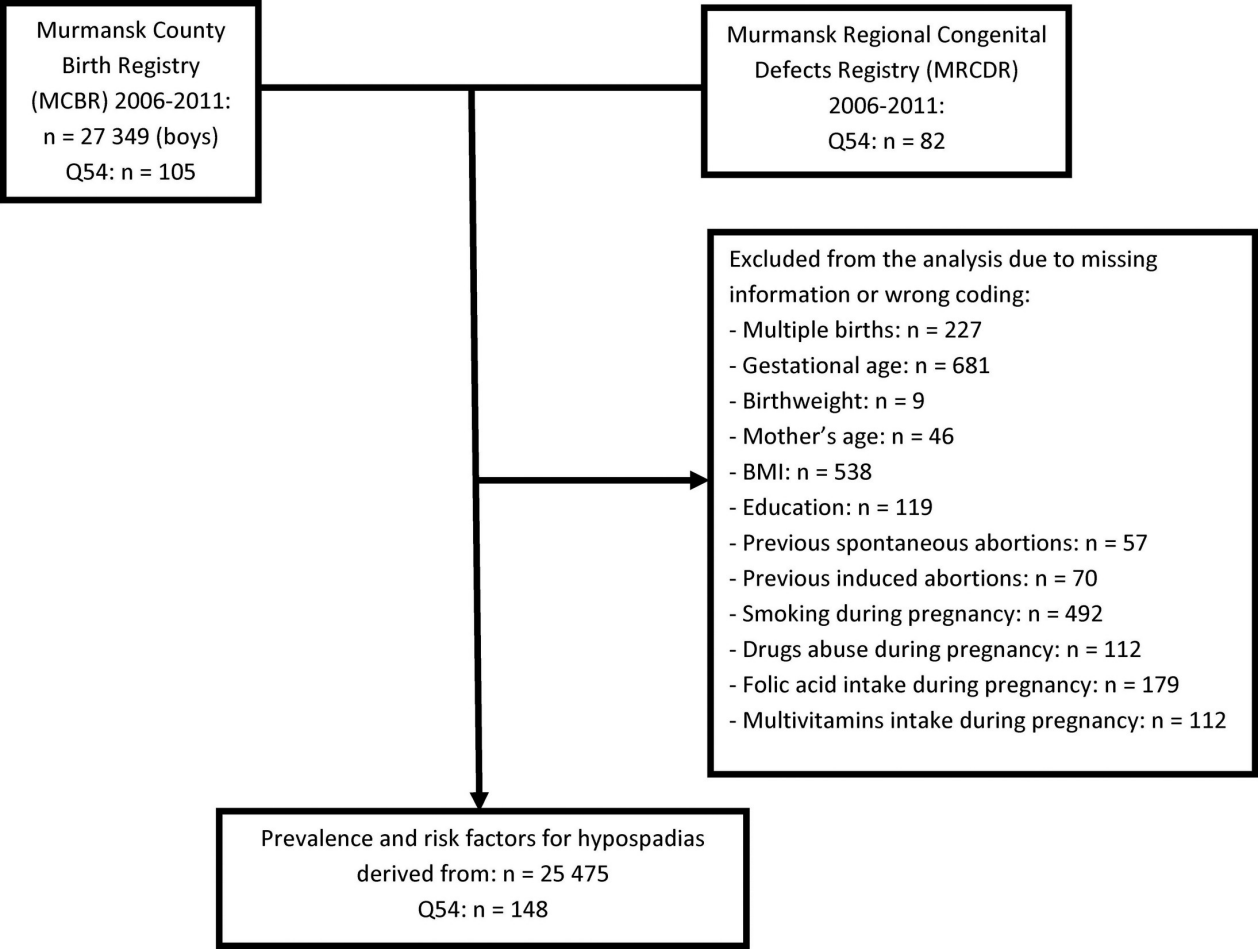
Number of births and exclusions for the combined Murmansk County Birth Registry and the Murmansk Regional Congenital Defects Registry (2006–2011). The individual numbers add up to more than the total number excluded due to missing information on two or more variables.

We included all male infants registered in the MCBR and MRCDR between 1 January 2006 and 31 December 2011. A detailed description of the MCBR has been published earlier and includes details about its implementation and quality control exercises [19]. The MRCDR has been in effect since January 1996 and includes data from week 28 of pregnancy to age 16. A diagnosis of hypospadias (International Classification of Diseases Revision 10 code Q54) depends on the location of the urinary opening (meatus). We combined information from the MBCR and MRCDR and removed duplicate records to obtain the final study sample. The manual merging of the data from the two registries was by the mother’s hospital ID number and birthdate, and the birthdate of the baby. Detailed descriptions of the MCBR and MRCDR as well as the linkage procedure have been published recently [18]. We included only singleton deliveries. After registry linkage, entries in the MCBR with missing information or erroneous coding (N = 1874) for selected variables were excluded from the study, which resulted in a final sample of 25 475 male infants (Fig 1).

### Statistical analyses

Isolated cases of hypospadias were selected and thus 148 cases were involved into analysis. Cases of mild, moderate and severe hypospadias as well as non-specified cases constituted one group; complex birth defects were not considered. We used Chi-square statistics to compare rates and the independent sample t-test for differences in mean values. Statistical significance was set at p ≤ 0.05. Logistic regression analysis was applied to identify risk factors associated with hypospadias, including neonatal birthweight and gestational age and the following maternal issues: residence, age at delivery, education, body-mass index at the first antenatal visit, parity, number of previous spontaneous and induced abortions, intake during pregnancy of progesterone-containing drugs, folic acid, multivitamins (not containing folic acid), hepatitis-B surface antigen (HBsAg) positivity, preeclampsia and alcohol/drug abuse.

Crude and adjusted odds ratios (ORs) with 95% confidence intervals (CI) were calculated. Variables that reached significance in the univariate analyses (namely, infant birthweight, preeclampsia) were included in the final multivariable logistic regression model. Cases of mild, moderate and severe preeclampsia constituted one group. In the final model, we included previously reported risk and protective factors for hypospadias (namely, maternal age at delivery, smoking during pregnancy, folic acid intake during pregnancy, and HBsAg positivity [20], and adjusted for gestational age. The statistical package IBM SPSS v.24.0 (IBM Corp., Armonk, NY, USA, 2016) was used for data analyses.

### Ethical considerations

Ethical approval was obtained from The Regional Health Administration of Murmansk County, the Ethics Committee of Gynecology-Obstetrician Association Group (reference number: 2013/14), Murmansk County, Russia, and the Norwegian Regional Committee for Medical and Health Research Ethics (ethical code reference number: 2013/2146). All data from the two registries were anonymized.

## Results

One hundred and five cases of hypospadias were registered in the MCBR and 82 in the MRCDR. After combining data from the two registries and removing duplicates, there were 148 cases of isolated hypospadias, corresponding to a total prevalence of 54.2 per 10,000 male births. Of the 148 only 110 cases were diagnosed during the perinatal period and the remainder within 3 months after birth. In terms of the ICD-10 classification of hypospadias and severity proportion, 84 cases (56.8%) belonged to the distal type of hypospadias (considered a mild form), 29 cases (19.6%) were of the midshaft type (moderate form), with 7 (4.8%) in the proximal group (a severe form) and 28 (18.8%) unspecified cases.

The mean birthweight was significantly lower (p < 0.01) in the group with hypospadias, while maternal age, the gestational age distribution, parity, previous induced and spontaneous abortions were comparable in both groups (Table 1). Multivitamin and folic acid intake were not significantly different in the two groups, while preeclampsia was higher among the cases (respectively, p = 0.03 and < 0.01). Additional details about the mothers and infants are provided in Table 1.

**Table 1:** Characteristics of cases and non-cases of isolated hypospadias (Q54). Data shown constitute a combined set for the Murmansk County Birth Registry and the Murmansk Regional Congenital Defects Registry during the period 2006–2011.

	Cases		Non-cases		
	N = 148^a^		N = 25 327^a^		
Variables	Value or	SD	Value or	SD	p-value^d^
	N^b^	*or*	N^b^	*or*	
		%^c^		%^c^	
**Infant Characteristics**					
*Birth weight (g)*, *mean ± SD*	**3291.0**	**540.7**	**3421.0**	**580.1**	<0.01
< 2500	14	9.5	1160	4.6	
2500–3999	121	81.7	20 899	82.5	
≥ 4000	13	8.8	3268	12.9	
**Maternal Characteristics**					
*Age at delivery (years)*, *mean ± SD*	**26.94**	**4.99**	**26.83**	**5.27**	0.79
< 20	6	4.1	1744	6.9	
20–34	130	87.8	21 338	84.2	
35+	12	8.1	2245	8.9	
Gestational age (weeks), mean ± SD	**39.5**	**1.9**	**39.4**	**2.2**	0.59
*BMI (kg/cm*^*2*^*)*, *mean ± SD*	**23.62**	**3.60**	**23.49**	**4.27**	0.72
< 18.5	11	7.4	1573	6.2	
18.5–24.9	92	62.2	16 567	65.4	
> 25	45	30.4	7187	28.4	
*Parity*					0.16
0	93	62.8	14 040	55.4	
1	48	32.4	9438	37.3	
≥2	7	4.7	1849	7.3	
*Previous induced abortions*					0.20
0	93	62.8	14 609	57.7	
≥1	55	37.2	10 718	42.3	
*Previous spontaneous abortions*					0.53
0	133	89.9	22 325	88.2	
≥1	15	10.1	2992	11.8	
*Education*, *years*					0.53
<12	55	37.2	8781	34.7	
12+	93	62.8	16 546	65.3	
Smoking during pregnancy	36	24.3	6264	24.7	0.91
Alcohol abuse during pregnancy	1	0.7	75	0.3	0.36
Drug abuse during pregnancy	0	0	85	0.3	0.61
Folic acid intake during pregnancy	108	73.0	18 832	74.4	0.70
Multivitamin intake during pregnancy	136	91.9	23 479	92.7	0.71
HBsAg positive	3	2.8	471	1.9	0.76
Preeclampsia	20	12.7	2171	8.6	0.03

^a^ Number of cases and non-cases are less than the entire study population due to missing values for some independent variables.

^b^ Means or numbers

^c^ Standard deviation (SD) or percentages

^d^ t-Test, Chi-square test or Fisher’s exact test

The crude and the adjusted ORs for the variables included in the logistic regression analysis did not differ substantially between cases and non-cases. Low infant birthweight was associated with a two-fold elevation of hypospadias risk in both the unadjusted and adjusted models (Table 2); and for preeclampsia, the increase was somewhat lower (OR values of 1.67 and 1.65, respectively). Smoking during pregnancy, folic acid intake during pregnancy and HBsAg positivity did not influence the risk of hypospadias.

**Table 2:** Crude and adjusted odds ratio (OR) with 95% confidence interval (CI) of isolated hypospadias^a^. Data shown constitute a combined set for the Murmansk County Birth Registry and the Murmansk Regional Congenital Defects Registry during the period 2006–2011.

	Crude		Adjusted^b^	
Variables	OR	95% CI	OR	95% CI
Birthweight (g)				
< 2500	2.09	1.20–3.64	2.06	1.18–3.60
2500–3999	1.00	Reference	1.00	Reference
> 4000	0.69	0.38–1.22	0.67	0.38–1.19
Age at delivery (years)				
< 20	0.57	0.25–1.28	0.55	0.24–1.25
20–34	1.00	Reference	1.00	Reference
35+	0.88	0.49–1.59	0.92	0.85–1.54
Smoking during pregnancy	0.98	0.67–1.42	0.97	0.66–1.42
Folic acid intake during pregnancy	0.93	0.65–1.34	0.92	0.64–1.32
HBsAg positive	1.09	0.35–3.44	1.02	0.32–3.23
Preeclampsia	1.67	1.04–2.68	1.65	1.03–2.66

^a^ Number of cases is 148 with 25 327 non-cases.

^b^ Each variable is adjusted for the others listed.

## Discussion

We found that low infant birthweight and preeclampsia were risk factors for hypospadias in Murmansk County, which suggest a linkage to changes in maternal hormone levels during early pregnancy. In agreement with our findings, previous reports suggest that alcohol consumption during pregnancy is not associated with the development of hypospadias [11, 21]. Similarly, the lack of an observed association between hypospadias and smoking during pregnancy has been reported [21–23]. Although high maternal age at delivery is suggested as a risk factor for hypospadias [24], most studies do not report such an association [25–28]. Our findings concur with the latter, and further illustrate that young maternal age at delivery does not influence the risk of having a son with hypospadias.

Therapeutic drugs such as corticosteroid hormones, antibiotics, or antifungal medications, are reported not to associate with hypospadias [29, 30], while the reported influence of progesterone-containing drugs varies [31]. Although oral contraceptives may cause high estrogen levels, limited association has been found between hypospadias and oral contraceptive use during pregnancy [32]. However, an experimental study in mice shows that high doses of synthetic estrogen during pregnancy induces hypospadias in 50% of male fetuses [33]. In humans, neither folate [34] nor iron supplementation [35] influence hypospadias risk [35, 36]. Although we did not have information on the use of all drugs and supplements taken during pregnancy, our logistic analyses indicate that folic acid intake was not associated with the risk of hypospadias.

Maternal hypertension during pregnancy and preeclampsia may associate with placental dysfunction, possibly by compromising utero-placental perfusion [36]. Weak spiral artery invasion of the placenta disturbs utero-placental perfusion during early gestation in women with gestational hypertension or preeclampsia [36]. Placental insufficiency may also affect fetal somatic and urethral development, and an association between hypospadias and low placental weight has been observed [37]. Since human chorionic gonadotropin (hCG) is a hormone produced by the placenta following implantation and placental hCG stimulates fetal testicular steroidogenesis, placental insufficiency may result in inadequate fetal hCG provision that leads to intrauterine growth retardation [38]. This may explain the association between hypospadias and low infant birthweight we and other researchers [39, 40] have observed.

### Strengths and limitations of the study

The high quality of data in the MCBR is a strength of this study. By combining MBCR and MRCDR, we can follow children up to 16 years of age, which helps identify more cases of hypospadias and other congenital malformations not diagnosed at birth.

This study may be limited through the inexperience of medical doctors to detect and correctly diagnose hypospadias, especially in remote areas of Murmansk County. This may contribute to systematic errors such as under reporting, over reporting, and misclassification of cases. A second limitation is that abortions before 22 weeks of gestation are not included in registries in Russia, and this hindered more accurate prevalence estimates. Our data on smoking, alcohol abuse, and drug abuse are in part self-reported, which may have led to underreporting. Another limitation is that severity of hypospadias was not analyzed. In our study for some of the variables we had 2.4% (Fig 1) of missing data and applied a listwise deletion method. These necessary exclusions from the logistic regression analysis might have biased our risk ratio estimates, which could have resulted in an underestimation of the risk ratios. One more potential limitation is that all three degrees of preeclampsia constituted a single variable. Finally, not all possible maternal, perinatal, and environmental risk factors were included in the analysis due to the relatively small sample size.

## Conclusion

Our Russian registry-based data showed that hypospadias was associated with low infant birthweight and preeclampsia in Murmansk County, Northwest Russia. The consistent association between hypospadias and low infant birthweight and preeclampsia suggests placental insufficiency.
